# Fabrication and appraisal of targeted axitinib loaded bilosomes for the enhanced breast and ovarian anticancer activity

**DOI:** 10.1371/journal.pone.0325511

**Published:** 2025-07-17

**Authors:** Randa Mohammed Zaki, Basmah Nasser Aldosari, Layla A. Alkharashi, Alyaa Alsalhi, Fatma I. Abo El-Ela, Raneem Meshal Alosaimi, Maha Alsunbul, Obaid Afzal, Mayada Said

**Affiliations:** 1 Department of Pharmaceutics, College of Pharmacy, Prince Sattam Bin Abdulaziz University, Al-Kharj, Saudi Arabia; 2 Department of Pharmaceutics and Industrial Pharmacy, Faculty of Pharmacy, Beni-Suef University, Beni-Suef, Egypt; 3 Department of Pharmaceutics, College of Pharmacy, King Saud University, Riyadh, Saudi Arabia; 4 Department of Pharmacology and toxicology, College of Pharmacy, King Saud University, Riyadh, Saudi Arabia; 5 Department of pharmacology, Faculty of Veterinary Medicine, Beni-Suef University, Beni-Suef, Egypt; 6 Department of Pharmaceutical Sciences., College of Pharmacy, Princess Nourah bint Abdulrahman University, Riyadh, Saudi Arabia; 7 Department of Pharmaceutical Chemistry, College of Pharmacy, Prince Sattam Bin Abdulaziz University, Al-Kharj, Saudi Arabia; 8 Department of Pharmaceutics and Industrial Pharmacy, Faculty of Pharmacy, Cairo University, Cairo, Egypt; University of Mashreq, IRAQ

## Abstract

The goal of this study was the formulation and optimization by statistical means of bilosomal formulations of axitinib (AXT) in order to improve its anticancer efficacy in a targeted manner. A central composite rotatable design was employed Using Design-Expert® software. The formulation factors were cholesterol, span 60, and sodium deoxy cholate (SDC) amounts (mg), whereas the dependent responses were Entrapment efficiency (EE%), Vesicles’ size (VS), and Zeta potential (ZP). The design expert software was utilized to perform the numerical optimization process. The optimized bilosomal formulation was assessed using differential scanning calorimetry (DSC), X-ray diffraction (XRD), transmission electron microscope (TEM), in-vitro release study, short-term stability study, and in-vitro cell proliferation assay and flow cytometry on MCF-7 breast and OV-2774 ovarian cancer cell lines. The optimized formulation was found to be composed of 19.999, 111.869 and 15 mgs of cholesterol, span 60, and SDC, respectively with a desirability of 0.753. EE%, VS, and ZP were predicted to be 88.4977%, 594.592 nm, and −44.2354 mV, respectively. The validation process on the optimized formula demonstrated that the variation from the predicted responses was less than 5%. The DSC and XRD studies revealed that AXT was entrapped within the bilosomal vesicles. The optimized AXT bilosomal formulation exhibited spherical non-aggregated nanovesicles in TEM images. Furthermore, it improved AXT release when compared to AXT suspension. According to stability experiments, the optimum bilosomal formulation was stable for thirty days. The cytotoxicity of the optimized bilosomal formulation was enhanced on the MCF-7 breast and OV-2774 ovarian cancer cell lines compared to AXT suspension even at lower concentrations. Flow cytometry showed that AXT loaded BSMs made a significant increase in the percentage of apoptotic cells in MCF-7 and OV-2774 cells, respectively. Molecular docking suggests that axitinib and SDC decreased the activation of the caspase-8 receptor on the surface of ovarian and breast cancer, which consequently led to an increase in anticancer activity. So, BSMs might be regarded a promising carrier of AXT to target ant treat breast and ovarian cancers.

## 1. Introduction

Cancer is a disease that is characterized by uncontrolled cell proliferation that originates in one region of the body and spreads to other organs and tissues, eventually leading to death. Early cancer detection and therapy are critical for limiting disease progression and lowering mortality rates [1,2]. Breast cancer is the most common cancer’s type in women [2]. As soon as discovered early and without metastases, it can be treated successfully in up to 80% of patients. Cancer patients with metastases, on the other hand, are difficult to treat with current medicines [3]. Breast cancer is characterized at the molecular level by a diverse array of genetic abnormalities, including mutations in epidermal growth factor receptor-2, (HER-2), hormone receptors, and other genes [4]. Chemotherapy and surgery are two frequent treatment options for breast cancer, relying on the molecular subtypes. Drug penetration, stability, and bioavailability are all limited, as with other solid tumors. Many ways have been proposed to address these issues by incorporating nanoparticles [5].

Ovarian cancer develops in female eggs and generally stays undiagnosed until it spreads to the stomach and pelvis [6]. These sorts of cancers in women are difficult to treat and, as a result, are nearly always fatal [7–9]. It is the second most prevalent female gynecological cancer [10]. Early menopause, as well as a high intake of saturated fats and processed carbohydrates, all raise the risk of ovarian cancer [11]. Vegetable eating, on the other hand, lowers the incidence of ovarian cancer [10].

Cancer development is a complicated process involving the activation of various signaling pathways for cell proliferation. Constant blood flow is essential to meet the high demands of developing cancer cells. Because fewer blood vessels reach the affected area as the tumor grows in size, the availability of oxygen and other nutrients to the core cells becomes reduced [12]. Furthermore, distinct angiogenesis-promoting signaling systems are triggered in the tumor location to ensure cancer cell survival. Angiogenesis is mediated by vascular endothelial growth factor (VEGF) after it interacts with VEGF receptors (VEGFR 1, 2, and 3). As a result, the development of a therapeutic strategy that includes small molecule inhibitors capable of efficiently exploiting the hypoxic tumor microenvironment by reducing angiogenesis could represent a substantial advancement in cancer treatment [12].

Axitinib (AXT) is a small molecule anticancer medicine that is tyrosine kinase receptor inhibitor of VEGFR1, 2, and 3 as well as platelet derived growth factor (PDGF) [12].

Currently available therapeutic options include radiotherapy, chemotherapy, immunotherapy, and surgery [13–15]. These alternatives are either invasive or have a history of toxicity, adverse effects, treatment resistance, and relapse [16]. Researchers and physicians are being asked to collaborate in order to enhance outcomes of the therapy. The use of nano drug delivery systems and safer drug moieties are important factors in developing anticancer treatments with fewer adverse effects [2,16].

Nanotechnology is now one of the most widely used methods in cancer research, having applications in cancer detection and treatment. Drug delivery [17], gene therapy, detection and diagnostics, drug transportation, biomarker mapping, targeted therapy, and molecular imaging are some of the anticipated consequences of nanotechnology. Nanomaterials, such as gold nanoparticles and quantum dots, have been produced using nanotechnology for molecular cancer diagnosis [1]. Biomarkers are an example of how nanotechnology-based molecular diagnostics may diagnose tumors fast and accurately. Biomarkers are an example of how nanotechnology-based molecular diagnostics may diagnose tumors rapidly and in a reliable manner [1]. The development of nanoscale drug delivery systems, for example, can allow precise targeting of cancerous tissue with minimum side effects [18]. Nanomaterials can easily circumvent cell barriers due to their molecular properties [19]. Due to its active and passive targeting, nanomaterials were employed for cancer therapy several years ago. While various drugs may be used for cancer treatment, their sensitivity frequently leads to poor outcomes, side effects, and injury to healthy cells. As a result, multiple studies have examined various forms of nanomaterials, such as liposomes, polymers, molecules, and antibodies, and found that incorporating these nanomaterials into the design of cancer treatments can improve medication efficacy while decreasing toxicity [1].

AXT was previously loaded into many Nano formulations for the treatment of cancer including nanohybrid liposomal nanoparticles [20], dual pH responsive micelle [21], PEGylated lipid bilayer supported mesoporous silica nanoparticles [12], pH-Activatable copper-axitinib coordinated multifunctional nanoparticles [22].

One of the most common difficulties in the treatment of cancer is to make targeting of the medications to defined action areas while reducing side effects and the resistance of the drug, that may be accomplished by overcoming drug efflux transporters [23]. Bilosomes (BSMs) have lately been employed as promising carriers for cancer treatment delivery since they increase drug targeting and reduce adverse effects. These novel nanocarriers are a bile salt base niosomal systems. They have high deformability and flexibility than liposomes and niosomes. The presence of bile salt in BSMs enhances the drug molecules permeability across biological barriers and increases the drug bioavailability [24,25]. In addition, the presence of bile salts in a nanocarrier system’s lipid bilayer provides a protection of the entrapped drug from different gastrointestinal fluids [26–29]. Based on all of these characters, BSMs have been used as a drug delivery system in various studies. They have been used for loading many drugs for the treatment of cancer like piceatannol loaded liposomes in lung cancer [30], TPGS surface modified bilosomes as boosting cytotoxic oral delivery systems of curcumin against doxorubicin resistant MCF-7 breast cancer cells [31], PEGylated Bilosomal Nano-Vesicles of Acrylamide for breast cancer [32], mucoadhesive surface modified bilosomes of psoralidin for breast and lung cancer [33], costunolide loaded bilosomes for the treatment of colon cancer [34], tamoxifen bilosomes for the treatment of breast cancer [35]. The materials used in BSMs formulation are widely known to be safe, biocompatible and biodegradable [36].

The aim of our work was to make formulation of targeted AXT-loaded BSMs and evaluate their ability to boost AXT’s anticancer activity. To that purpose, we employed a central composite rotatable statistical design to assess the effect of various concentrations of cholesterol, span 60, and SDC on the entrapment efficiency (EE%), Vesicle size (VS), and Zeta potential (ZP) of the BSMs. The optimum bilosomal formula was then chosen and characterized with respect to DSC, XRD, transmission-electron microscopy (TEM), in-vitro release, short term stability, molecular docking, in-vitro cell proliferation assays, and flow cytometry on MCF-7 breast and OV-2774 ovarian cancer cell lines.

## 2. Materials and methods

### 2.1. Materials

Axitinib (AXT), cholesterol, span 60, Sodium deoxycholate (SDC), Methanol, and chloroform were all purchased from Sigma Aldrich (St. Louis, MO, USA). Breast cancer cells (MCF-7) and ovarian cancer cells (OV-2774) were obtained from ATCC. DMEM/F2, antibiotic and antimycotic solutions were obtained from GIBCO®. Invitrogen TM, containing 10% FBS and Universal Mycoplasma Detection Kits (ATCC 30‐1012K) were purchased from Sigma-Aldrich.

### 2.2. Statistical design of AXT loaded BSMs

Using a central composite rotatable design, the effect of several variables of the formulation on the entrapment efficiency (EE%) (Y1), Vesicle size (VS) (Y2), and Zeta potential (ZP) (Y3) of AXT loaded BSMs was investigated. Design Expert® software from Stat-Ease (Ver. 12, Minneapolis, Minnesota, USA) was used. The cholesterol amount (A) was set between 10 and 20 mg, the Span 60 amount (B) between 50 and 150 mg, and the SDC (X3) between 5 and 15 mg. In all formulations, the AXT concentration was kept constant at 0.1 (%w/v). This resulted in 20 experimental runs. Table 1 displays the formulation variables (low and high level) and dependent responses. Table 2 shows the composition of AXT-loaded BSMs.

**Table 1:** Central Composite Rotatable Design for optimization of AXT loaded BSMs.

Independent variable.	Levels				
**Low**			**High**	
Cholesterol amount (mg) (A)	10			20	
Span 60 amount (mg) (B)	50			150	
SDC amount (mg) (C)	5			15	
**Dependent variables**	R2	Adjusted R2	Predicted R2	Constraints	Adequate precision
EE% (Y1)	0.9022	0.8839	0.8337	Maximize	22.8909
Vesicles size (nm) (Y2)	0.9663	0.9359	0.7652	Minimize	19.9379
Zeta potential (mV) (Y3)	0.9558	0.9160	0.7225	Maximize	18.6803

**Table 2:** Output data of Central Composite Design for optimization of AXT loaded BSMs.

Formula code	Independent variables			Dependent variables			
Cholesterol amount (mg) (X1)	Span60 amount(mg) (X2)	SDC amount (mg) (X3)	EE% (Y1)	Vesicles size (nm) (Y2)	Zeta potential (mV) (Y3)	PDI
BSMs1	15	184.09	10.5	87.2 ± 1.56	980.4 ± 15.6	−38 ± 1.21	0.645 ± 0.03
BSMs2	10	150	15	81 ± 2.02	744.1 ± 8.97	−45.6 ± 1.32	0.543 ± 0.05
BSMs3	10	150	6	78.8 ± 2.46	819.1 ± 6.87	−34.5 ± 0.93	0.363 ± 0.12
BSMs4	15	100	10.5	83.7 ± 0.98	570.7 ± 5.76	−38.4 ± 0.76	0.267 ± 0.04
BSMs5	15	100	10.5	82.2 ± 2.24	508.4 ± 4.89	−37.6 ± 0.54	0.274 ± 0.07
BSMs6	15	100	2.93193	80.2 ± 3.5	485.5 ± 5.23	−37.1 ± 0.46	0.204 ± 0.15
BSMs7	20	50	15	84.7 ± 1.89	667.4 ± 6.34	−46.2 ± 1.42	0.328 ± 0.17
BSMs8	23.409	100	10.5	89.6 ± 1.42	746.7 ± 8.75	−39.5 ± 0.71	0.435 ± 0.05
BSMs9	10	50	15	74.8 ± 1.78	429.9 ± 8.21	−42.1 ± 0.82	0.432 ± 0.10
BSMs10	6.59104	100	10.5	76.2 ± 2.65	567.1 ± 9.41	−36.8 ± 0.48	0.573 ± 0.02
BSMs11	20	150	15	91.4 ± 1.44	763.1 ± 8.87	−44.6 ± 1.28	0.576 ± 0.01
BSMs12	20	50	6	83.11 ± 2.38	849.3 ± 8.85	−39.8 ± 0.34	0.489 ± 0.011
BSMs13	15	100	10.5	80.2 ± 2.53	505.6 ± 7.12	−37.0 ± 0.74	0.365 ± 0.13
BSMs14	10	50	6	74.2 ± 1.46	649.3 ± 7.84	−35.3 ± 1.28	0.454 ± 0.14
BSMs15	15	100	10.5	81.9 ± 2.11	504.8 ± 6.94	−37.2 ± 1.63	0.334 ± 0.06
BSMs16	20	150	6	89.5 ± 1.98	593.9 ± 5.11	−37.6 ± 1.03	0.278 ± 0.14
BSMs17	15	100	10.5	82.8 ± 3.02	511.2 ± 9.56	−36.2 ± 0.67	0.540 ± 0.04
BSMs18	15	15.9104	10.5	70.1 ± 2.82	976.0 ± 9.83	−36.5 ± 0.75	0.588 ± 0.01
BSMs19	15	100	18.0681	84.7 ± 1.38	433.0 ± 6.11	−48.3 ± 0.33	0.287 ± 0.03
BSMs20	15	100	10.5	83.6 ± 1.63	512.4 ± 5.34	−38.1 ± 0.88	0.269 ± 0.05
Optimized formula	19.999	111.869	15				

### 2.3. Preparation of AXT loaded BSMs

AXT BSMs were prepared using modified ethanol injection method [37]. In brief, 0.01 gm AXT and the specified amounts of cholesterol and span 60 were dissolved in 10 ml methanol and chloroform mixture 1:1 (v/v). SDC was dissolved in 10 ml distilled water. The organic phase was added drop wise to the aqueous phase which was previously heated to 60 ͦC. The stirring was continued on the hot magnetic stirrer at 60 ͦC and 800 rpm for half an hour till the organic solvent was completely evaporated.

### 2.4. Characterization of AXT loaded BSMs

#### 2.4.1. Determination of entrapment efficiency (EE%).

A cooling centrifuge (SIGMA 3–30 K, Sigma, Steinheim, Germany) at 17000 rpm for 1 hour at 4 ͦC separated AXT-loaded BSMs from the free drug [38,39]. AXT in the supernatant was quantified using a UV spectrophotometer (Shimadzu UV-1800, Kyoto 604–8511, Japan) after suitable dilution. The measurements were done at λ_max_ (259 nm). Within the concentration range of 4–16 µg/ml, the technique was validated for linearity (R2 = 0.997).

The EE% was measured through the subsequent equation [39]:


$$EE\%=\frac{TD-FD}{TD}\times 100\;$$ (1)

Where FD denotes the amount of free drug, TD the total amount of drug, and EE% the percentage of entrapment efficiency.

#### 2.4.2. Determination of vesicle size (VS), polydispersity index (PDI), and zeta potential (ZP).

The VS, PDI, and ZP values were measured using a Zetasizer (Malvern Instruments, Worcestershire, UK). After adequate dilutions with distilled water, the measurements were performed at 25 degrees Celsius [38]. Every measurement was taken three times.

### 2.5. Statistical analysis, optimization, and validation

To establish statistical significance of the observed responses, a statistical factorial analysis of variance (ANOVA) was done by applying the Design Expert® software. The optimum formula with the highest EE% and ZP and the lowest VS was selected using a desirability function was. Then, it was prepared and assessed with respect to EE%, VS, and ZP to make validation of the statistical models used. The percentage errors among the expected values and the obtained results were then calculated [39,40]:


$$\%\;Relative\;error\;=\;\frac{values\;predicted-obtained\;findings}{values\;predicted}\times \;100\mathrm{\backslash ]}$$ (2)

### 2.6. Evaluation of the optimum AXT loaded BSMs

#### 2.6.1. Transmission electron microscopy (TEM).

A transmission electron microscope (TEM; JEOL JEM-1010, Tokyo, Japan) was used to examine the morphology of the optimized AXT formulation. For this aim, samples were diluted and placed on a carbon-coated copper grid. They were then coated with 2% (w/v) phosphotungestic acid, air dried for 5 minutes, and photographed using a TEM at room temperature with an X80000 magnification power and an acceleration voltage of 80 KV [41].

#### 2.6.2. Study of *in-vitro* release.

For comparing the release of AXT from the optimum formula and AXT suspension, the equivalent of 5 mg of each was placed inside dialysis bag. Following that, both were suspended in 250 mL of dissolving media (phosphate buffer pH (6.4)) [42] in a dissolution device (Pharm Test, Hainburg, Germany) set at 37 ͦC and 100 rpm stirring. The dissolution media was then sampled at 1, 2, 3, and 4 hr. intervals, and an equivalent volume of fresh media was immediately added. A UV spectrophotometer was used to determine the concentration of AXT in the obtained samples. The following calculation was used to compute the proportion of AXT released at various time points [38]:


$$Qn=\;\frac{Cn\;x\;Vr+\;\sum_{i=1}^{n-1}Ci\;x\;Vs}{initial\;drug\;content}\mathrm{\backslash ]}$$ (3)

Where,

Qn: Cumulative percentage of AXT released

Cn: The dissolution medium AXT concentration at the n^th^ sample

Vr: The dissolution medium volume

Vs: The volume of the sample

$\sum_{i=1}^{n-1}Ci\backslash )$: The sum of the previously measured concentrations

A plot of the proportion of AXT released (Qn) at different time points vs. the relevant time was developed to define the release profile of the optimized AXT-loaded BSMs formulation compared to the AXT suspension.

#### 2.6.3. Studying the aging effect.

The optimized AXT-loaded BSMs formulation stability was evaluated at various time intervals for thirty days in an airtight container kept away from light at 4° C. The withdrawn samples were assessed in terms of EE%, VS, and ZP [38,39,43–45].

#### 2.6.4. Differential scanning calorimetry (DSC).

DSC study was performed on free AXT and the optimum BSMs formula. DSC investigations were carried out using a differential scanning calorimeter ((DSC N-650; Scinco, Italy)) by inserting around 5 mg of material in its aluminium pan and heating it to 300° C at a rate of 10° C/min in a dry nitrogen atmosphere.

#### 2.6.5. Study of X-ray diffraction (XRD).

An Ultima IV Diffractometer (Rigaku Inc. Tokyo, Japan College of Pharmacy, King Saud University, Riyadh, KSA) was used to scan the X-ray diffraction patterns of free AXT and the optimum BSMs formula at 10°/min speed rate and 0–60° range

### 2.7. In-vitro anticancer activity studies

#### 2.7.1. Cells and cell culture.

Breast cancer cells (MCF-7) and ovarian cancer cells (OV-2774) were obtained from ATCC, authenticated by ATCC using short tandem repeat profiling, propagated, expanded, and frozen immediately into numerous aliquots after arrival. The revived cells were used within 10–12 passages and for a maximum of three months after their revival. The cells were grown in DMEM/F2 (1:1) from GIBCO®, Invitrogen TM, containing 10% FBS and 1% antibiotics/antimycotic. Detection of mycoplasma contamination has been carried out regularly using Universal Mycoplasma Detection Kits (ATCC 30‐1012K). Sigma-Aldrich provided all supplements except for the antibiotic and antimycotic solutions, which were obtained from Gibco. The cells were maintained at 37°C in a humidified incubator containing 5% CO2.

#### 2.7.2. Cell proliferation assay, WST-1.

The optimum AXT BSMs formulation was compared to the free AXT suspension using the two cell lines mentioned above using the WST1 assay (Sigma-Aldrich). 5000 cells were seeded into 96-well microtiter plates in a final volume of 100 Liters of a suitable culture medium and incubated overnight. 0.4-fold serial dilutions were added to plain, AXT free dug and BSMs formulation (0.4–1.6 µM) on MCF-7-, and 10-fold serial dilutions (10–40 μM) were added to OV-2774 for an additional 48 hours. After cell treatment, WST1 reagent (ab155902) was added and incubated for four hours at 37°C. In the control wells, 100 µl of culture medium were combined with 10 µl of WST-1. ELISA microplate readers (Thermo Fisher Scientific, USA) were used to measure the intensity of formazan dye at 440 nm. In order to calculate the cytotoxicity percentage, the following formula was used;


% Cytotoxicity=((100*(control−treated sample)))/Control\] (4)

#### 2.7.3. Apoptosis measurement by flow cytometry.

An Annexin V Apoptosis Detection Kit (Multisciences, Hangzhou, China) was utilized to measure apoptosis of MCF-7 and OV-2774 cells. After treatment with the determined IC50 dose for 72 h, cells were trypsinized and washed with PBS, and resuspended in the binding buffer at a concentration of 106 cells/ml. Subsequently, 5 μl of Annexin V-FITC and 5 μl of propidium iodide (PI) were added and vortexed gently. Cells were then and incubated for 15 minutes at room temperature. Apoptotic rate was analyzed using flow cytometry (NovoCyte, USA) and the data was analyzed using NovoExpress software.

### 2.8. Molecular docking

#### 2.8.1. Ligand and protein receptor preparations.

SDC and axitinib were examined for 3D and 2D binding affinity and bonding against the predicted protein on breast and ovarian cancer cells. PubChem provided Axitinib’s 3D SDF X-ray crystal structure (https://pubchem.ncbi.nlm.nih.gov/compound/3086069).

a homology model of caspase-8 was provided by the protein data bank and Uni-prot (https://www.uniprot.org/uniprotkb/Q12906/entry). https://www.genecards.org/cgi-bin/carddisp.pl?gene was used to verify the human gene bank code and ID. Following the identification of the active site or pockets of interactions from the CB. DOCK2 site and the Deep site, these existing structures were employed as mooring targets. The quality of proteins was enhanced by ModRefiner (https://zhanggroup.org/ModRefiner/) through the enhancement of 3D model structures. The active sites were predicted by Deep Site Server (https://www.deepsite.ai/) and verified more accurately by CB. DOCk2 (https://cadd.labshare.cn/cb-dock2/php/blinddock.php#job_list_load).

#### 2.8.2. Computations and ligand preparation.

The ligands, Axitinib and SDC, were obtained in 3D-sdf format using the PubChem database. The energy-minimization procedure on their 3D structures was executed using the Avogadro 1.2.0 software 25 and an MMFF94 force field. The protein preparations will be followed by the analysis of the ligand, which is currently stored in pdb format, on Auto Duck Vienna. By conducting this analysis in pdbqt, the ligand will be primed for visualization and interactions on the Biovia 2021 program.

#### 2.8.3. Receptor preparations.

In order to produce proteins, a three-dimensional (3D) model of the diverse receptors was acquired. The PDB structure that Uni-prot has downloaded contains the entire protein’s alpha fold structure, XRD, and all chain types. The distinctive protein was synthesized by removing water molecules and introducing hydrogen atoms to the unoccupied valences of the heavy atoms prior to docking. The protein’s structure was analyzed to determine if any atoms were missing and necessitated the addition of KOLLMAN charges. Any sections that were incomplete were rectified. The Define and Edit Binding Site protocol, in conjunction with the deep site and CB-DOCK2 server, was employed to identify the appropriate binding site (Pockets), which includes active sites of interaction with the carvacrol ligand. The proteins were classified as receptors. The docking simulations were conducted on a grid frame of 30 × 30 × 30 using the Auto Dock Vina software. The active site was estimated and determined by employing a grid box that measured 30 x 30 x 30 along the x, y, and Z axes. The grid box was subsequently centered at the exact center of the active site. Subsequently, the precise amino acids obtained from the CB-Dock2 were employed to identify the active sites. All of the following are in the A-chain: Caspase-8; L: 254, L: 257, R: 260, D: 319, C: 360, D: 363). Once they were verified, the sites that were identified as active were eliminated. In the configuration file, the binding affinity between the ligand and receptor was determined, and the grid box’s dimensions were recorded for future reference. Ultimately, the protein was encoded in pdbqt format to facilitate docking with the ligand using the visualization DISCPVERY STUDIO (BIOVIA 2021) program.

#### 2.8.4. Interactions of ligands and receptor.

The protein–ligand interactions were visualized and analyzed using the server software after the binding affinity between the ligand and receptor was determined. Subsequently, the ligand was designated as Axitinib and SDC in pdbqt format. The receptor was introduced in pdbqt format and designated as a diverse array of proteins. The receptor was designated for each protein twice: once with the ligand and again with the standard medication. The ligand and receptor’s interactions were ascertained by integrating all amino acids, angles, and atom distances in the desired format. The following interactions were investigated: H-bonding, hydrophobic interaction, salt bridge between covalent bonds, aromatic ring center, charge center, and π-stacking (parallel and perpendicular). The protein–ligand interaction was evaluated by calculating the binding affinity and free energy (ΔG). In order to investigate interactions, all interactions were recorded in both 3D and 2D at varying dimensionalities.

## 3. Results and discussion

### 3.1. Evaluation of AXT loaded BSMs

#### 3.1.1. Estimation of EE%.

As exposed in Table 2, the EE% of several bilosomal formulae varied between 70.1282% to 91.4144%. S1 Fig demonstrates the effect of cholesterol amount (mg) (A), Span 60 amount (mg) (B), and SDC amount (mg) (C) on the EE%.

The linear model was in a good fit to EE% data (p-value < 0.0001), where the lack of fit was not statistically significant (p-value = 0.1627). The difference among the adjusted and predicted R2 was less than 0.2, indicating that the model was valid [38,39,46]. The results reveal that the model had adequate precision 22.8909, implying that it could work well within the planned area [47,48] as shown in Tables 1 and 3.

**Table 3:** ANOVA of central composite design for AXT loaded BSMs.

Dependent Variable	Source	SS	Df	Mean Square	F value	p value	
Y1	Model	502.56	3	167.52	49.23	< 0.0001	significant
	A-cholesterol amount	285.53	1	285.53	83.91	< 0.0001	
	B-Span 60 amount	202.97	1	202.97	59.64	< 0.0001	
	C-SDC amount	14.06	1	14.06	4.13	0.0590	
	Lack of Fit	46.03	11	4.18	2.48	0.1627	not significant
Y2	Model	5.339E + 05	9	59317.24	31.84	< 0.0001	significant
	A-cholesterol amount	20829.26	1	20829.26	11.18	0.0074	
	B-Span 60 amount	8056.38	1	8056.38	4.32	0.0642	
	C-SDC amount	11447.48	1	11447.48	6.15	0.0326	
	AB	51793.71	1	51793.71	27.80	0.0004	
	AC	9919.36	1	9919.36	5.32	0.0437	
	BC	30690.03	1	30690.03	16.47	0.0023	
	A²	28791.98	1	28791.98	15.46	0.0028	
	B²	3.611E + 05	1	3.611E + 05	193.85	< 0.0001	
	C²	9138.83	1	9138.83	4.91	0.0511	
	Lack of Fit	15357.69	5	3071.54	4.70	0.0574	not significant
Y3	Model	278.62	9	30.96	24.01	< 0.0001	significant
	A-cholesterol amount	17.01	1	17.01	13.19	0.0046	
	B-Span 60 amount	0.1482	1	0.1482	0.1150	0.7416	
	C-SDC amount	184.06	1	184.06	142.76	< 0.0001	
	AB	5.28	1	5.28	4.10	0.0705	
	AC	2.53	1	2.53	1.96	0.1914	
	BC	3.00	1	3.00	2.33	0.1581	
	A²	3.87	1	3.87	3.00	0.1138	
	B²	0.5771	1	0.5771	0.4476	0.5186	
	C²	65.20	1	65.20	50.57	< 0.0001	
	Lack of Fit	9.72	5	1.94	3.07	0.1219	not significant

The equation below demonstrated how the EE% was affected by the variables of formulation


EE%=81.9955+4.5725 A+3.85511 B+1.01473 C \] (5)

The ANOVA analysis in Table 3 reveals that cholesterol amount(mg) (A) and Span 60 amount (mg) (Bhad a significant effect on the EE% values (p-values < 0.0001). while SDC amount (mg) (C) didn’t show any significant effect on EE% values. Concerning the effect of cholesterol amount(mg) on EE%, increasing its amount led to a significant increase in EE% which could be related to the increased hydrophobicity and firmness of the lipid bilayer membrane. This stabilizes the BSMs and prevents AXT leakage from them [49]. These results complies with those of Saifi et al [24] who made a study on the effect of the cholesterol concentration on the EE% of acyclovir loaded BSMs.

Similarly, increasing the surfactant amount (span 60) significantly increased the EE%, probably due to the high phase transition temperature of span 60 (50 ͦC) in addition to the presence of long alkyl chain which increases the solubility and permeability of AXT in the lipid bilayer of BSMs with the result of increasing the EE% [50]. Also, increasing the amount of span 60 reduces the fluidity of the BSMs membrane and, as a result, lowered AXT leakage, thereby increasing the EE% [51]. These results agree with that of Ameeduzzafar et al [49] who studied the effect of span 60 concentration on the EE% of apigenin loaded oral nano-bilosomes.

#### 3.1.2. Evaluation of VS, PDI and ZP.

The VS of several BSMs formulations was in the range of 433.0 ± 6.11 to 980.4 ± 15.6nm, as shown in Table 2. S2 Fig depicts the effect of cholesterol amount (mg) (A), Span 60 amount (mg) (B), and SDC amount (mg) (C) on VS.

The quadratic model suited the VS data the best (p-value < 0.0001). A non-significant lack of fit (p-value 0.0574) and a minor discrepancy among the adjusted and predicted R2 (<than 0.2) confirm the model’s validity [38,39,46]. As indicated in Tables 1 the adequate precision was 19.9379 (higher than 4), showing that the model could work well within the planned space [46,48].

The preceding equation illustrates the impact of the variables of the formulation on VS:


$$\begin{array}{cc} Vesicles\;size & =+519.479+39.0536\;A+24.2882\;B-28.9521\;C-80.4625\;AB\;+35.2125\;AC+61.9375\;BC\\ & +44.6976\;A^{2}+158.294\;B^{2}-25.1822\;C^{2} \end{array}$$

ANOVA analysis (Table 3) pointed out that cholesterol amount (mg) (A), and SDC amount (mg) (C) significantly affected VS (p-values = 0.0074 and 0.0326) respectively. On the other hand, span 60 amounts (mg) (B) didn’t show any significant effect on VS values.

Concerning the effect of cholesterol amount (mg) on VS, it was found that increasing its concentration led to a significant increase in VS of BSMs which could be related to inhibiting the compact packing of lipid vesicles, resulting in increased aqueous phases within the bilosomes vesicle and an increase in vesicle size [49]. Another reason could be attributed to the increase in the EE% of AXT inside the bilosomal vesicles with the increase of cholesterol concentration which led to an increase in the size of the formed BSMs [24]. These results are in compliance with Ameeduzzafar et al [50] who studied the effect of cholesterol on the VS of chitosan polymeric vesicles of ciprofloxacin.

With regard to the effect of SDC concentration on VS, it was found to decrease the VS of BSMs significantly which could be related to the decrease in the surface tension and interfacial tension among the vesicle bilayer subsequently the space between the bilosomal vesicle bilayer [52,53]. These results complies with that published by Zafar et al [49] who made a study on the effect of bile salt in the VS of Apigenin loaded oral nano bilosomes.

The PDI refers to the variance in size between particles and lies between 0–1 [48]. Table 2 pointed out that the PDI values of the BSMs formulae varied between 0.204 ± 0.15 to 0.645 ± 0.03, suggesting that they were within the permitted size range [46].

The physical stability of the produced formulations is highlighted by ZP. Increasing the ZP value raises the repulsion between the vesicles, resulting in a decrease in aggregation and increased system stability [48]. Formulations with zeta potential values less than −30 or greater than +30 are extremely stable [54].

The ZP of the developed BSMs formulations was set between −48.30.33 and −34.50.93 mV (Table 2), confirming that the BSMs formulations are physically stable [39]. S3 Fig depicts the effects of cholesterol amount (mg) (A), Span 60 amount (mg) (B), and SDC amount (mg) (C) on ZP.

The ZP data were best fitted to a Quadratic model (p value < 0.0001) with an adequate precision of 18.6803 and a little discrepancy among adjusted and predicted R2 (Table 1). The succeeding equation demonstrates the impact of variables of formulation on ZP:


$$\begin{array}{cc} ZP & =-37.377-1.11598\;A-0.104174\;B\;-3.67113\;C+0.8125\;AB+0.5625\;AC\;-0.6125\;BC\\ & -0.518314\;A^{2}-0.200116\;B^{2}-2.12698\;C^{2} \end{array}$$

The values of the zeta potential values are affected in a significant way by cholesterol concentration (A) (p-values = 0.0046) and SDC concentrations (C) (p-values < 0.0001).

Cholesterol increase significantly increased the absolute values of the ZP of BSMs which could be explained by the introduction of negative charge into the BSMs surfaces which could be related to the uneven polarity distribution of cholesterol hydroxyl groups [55,56]. Our findings agree with that of Ismail et al [57] who made a study on the effect of cholesterol concentration on the ZP of sertraline hydrochloride bilosomes.

For SDC, its increase led to a significant increase in the absolute values of the ZP which could be related to its ionic nature. It imparts negative charge on the vesicles’ surfaces [48]. This was in agreement with the results published by Al-Mahallawi et al [28]who studied the effect of SDC concentration on the ZP of tenoxicam bilosomes.

### 3.2. Statistical analysis, optimization and validation

A numerical optimization was conducted using Design Expert® software to explore an ideal BSMs formulation while minimizing VS, ZP, and maximizing EE%. This resulted in an optimal BSMs formulation composed of 19.999 mg of cholesterol, 111.869 mg of span 60, and 15 mg of SDC with a desirability of 0.753 as shown in S4 Fig Table 4 and Fig 4 shows the expected values for EE%, VS, and ZP, which were 88.4977%, 594.592 nm, and −44.2354 mV, respectively. As exposed in Table 4, the optimal formula was prepared and validated, with a relative error of less than 5% from the predicted outcomes produced by the Design Expert software, indicating model fitness [38,39,46–48].

**Table 4:** Validation of the optimum formula.

Responses	Predicted value	Experimental value	% Relative error
EE%	88.4977	90.54	2.308
Vesicles size	594.592	610.43	2.664
Zeta potential	−44.2354	−45.67	3.243

### 3.3. Evaluation of the optimum BSMs formula

#### 3.3.1. Transmission electron microscopy (TEM).

As shown in S5 Fig, TEM imaging revealed spherical vesicles. There were no aggregates observed, which might be explained by the vesicle surfaces’ relatively high ZP, which caused repulsion of neighbouring BSMs [58].

#### 3.3.2. Study of the *in-vitro* release.

S6 Fig depicts the release profile of the optimal bilosomal formula compared to AXT suspension. When compared to the drug suspension, the optimal formula released more AXT. The reduction in vesicle size of the bilosomal formulation may resulted in drug release improvement [38]. The size of the vesicle affected drug release from nanovesicles, smaller vesicles resulted in a faster release rate than larger-sized ones [59,60]. In addition, the use of span 60 and SDC in bilosome formation led to the entrapment of AXT inside the bilsomes in a solubilised form.

#### 3.3.3. Study of the influence of aging.

The stability of the optimum BSMs formula after 30 days of storage is presented in Table 5 and S7 Fig The EE%, VS, and ZP did not show significant changes during the period of the experiment (7 and 30 days), suggesting that the optimized BSMs formulation was stable physically throughout storage at 4°C [38,39].

**Table 5:** Stability study for the optimized formula for one month at 4°C.

Responses	Fresh	After 30 days
EE%	90.54 ± 2.56	88.43 ± 2.78
Vesicles size (nm)	610.43 ± 8.45	618.87 ± 10.45
Zeta potential (mv)	−45.67 ± 1.12	−44.54 ± 1.17

#### 3.3.4. Differential scanning calorimetry (DSC).

The physicochemical properties and thermal behavior of pure AXT, cholesterol, span60, SDC, and AXT physical mixture, and AXT loaded BSMs were studied using DSC (S8 Fig). Because the physical state of drug molecules in nano-formulations affects their release and solubility in external medium, DSC thermograms are useful in determining the nature or physical state of the drug molecules alone and while being loaded in nano formulations [61]. AXT exhibited a pronounced endothermic peak at roughly 230 ͦ C [62], confirming its specific melting point (S8 Fig A). The drug’s endothermic peak was well retained in its physical mixture with cholesterol, span 60 and SDC with alterations in the form of broadening or moving the melt temperature (S8 Fig B). The amount of materials utilized, particularly in drug-excipient mixtures, may influence the enthalpy and form of the peak. So, these little changes in the drug’s melting endotherm may be due to mixing the drug with the excipients, which resulted in a reduction in the purity of the mixture’s individual components, and this may not imply a probable incompatibility [38,39]. As a result, the compatibility of AXT with formulation excipients may be deduced. The endothermic peak of AXT did not appear in the DSC spectra of AXT-BSMs (S8 Fig C), demonstrating the complete loading of AXT in BSMs in an amorphous state [38,39].

#### 3.3.5. Study of X-ray diffraction (XRD).

S9 Fig shows the XRD spectra of pure AXT, physical mixture of AXT, cholesterol, span 60 and SDC and the optimized AXT loaded BSMs formulation. In the XRD spectrum of pure AXT, sharp peaks at 2θ: 8.8, 9.4, 11.9, 14.5, 15.1, 15.6, 19.0, 19.3, 20.3, 20.6, 21.6, 23.1, 24.1, 24.6, 24.9 and 26.0 were observed, confirming crystallinity (S9 Fig A). The XRD spectrum of the physical mixture of AXT with cholesterol, span 60, and SDC demonstrated persistence of AXT peaks, indicating that AXT is compatible with the additives utilized (S9 Fig B) [39]. The entrapment of AXT inside BSMs vesicles resulted in reduction of the intensity of some drug peaks and the disappearance of others (S9 Fig C) [2]. Furthermore, the reduction in the intensity of AXT peaks shows that AXT is entrapped in an amorphous state inside the BSMs [2].

## 4. In-*vitro* anticancer activity studies

### 4.1. Cell proliferation assay, WST-1

We have evaluated AXT BSMs optimum formula with AXT free drug for different dosages on two human cancer cell lines (MCF-7 and OV-2774). The growth inhibitory effects of AXT BSMs formula were concentration-dependent in both cell lines (S10 Fig). The AXT BSMs showed enhanced anticancer activity of AXT on both cancer cell lines in comparison to AXT free drug suspension. The WST-1 assay revealed a reduction in cell viability percentage that was concentration dependant. Fig 10 showed a sharp reduction in the percent cell survival reaching 50% at a concentration of 0.7 µM for BSMs dosage form while the AXT free drug suspension made 50% cell survival at 1.1 µM concentration in breast cancer (MCF-7) cell line. On the other hand, only the AXTBSMs formula showed a 50% reduction in the cell survival at a concentration of 36 µM not AXT free drug suspension in ovarian cancer (OV-2774) cell line. This points out that the AXTBSMs formula was more effective in reducing 50% cell viability than the AXT free drug suspension. This might be due to the enhanced cellular uptake of AXTBSMs formula compared to the AXT free drug suspension.

### 4.2. Apoptosis measurement by flow cytometry

The percentages of apoptotic and necrotic cells in MCF-7 and OV-2774 cell lines (S11 Fig) were analysed by flow cytometry. Incubation with either 0.7 μM, or 36 μM of AXT BSMs nano formula for 72 h significantly increased the percentage of apoptotic cells in MCF-7 and OV-2774 cells, respectively (S11 Fig A–C). The % of apoptotic cells induced by AXTBSMs nano formula relative to free drug suspension was (74.79% vs. 55.15%) in MCF-7 and (52.2 vs 38.23) in OV-2774. This clearly shows that AXTBSMs is more effective than free AXT in suppressing tumour growth and survival in human cancer cells.

## 5. Molecular docking

### 5.1. Docking simulation

The docking experiments demonstrated that the predicted active sites of the tested receptors within the breast and ovarian carcinoma exhibited the highest binding affinity or scoring, as indicated by a higher negative energy requirement. In S12 Fig, the Uni-prot server was utilized to obtain the chemical structure and sequencing of the five proteins, as well as their recorded ID. After downloading the proteins in pdb format, the CB-Dock2 server was implemented to identify the active sites of targeting for interaction. Besides the amino acids that were shared, S13 Fig illustrate that the Figs contained compartments. In the end, the grid box dimensions for each target protein were determined, as illustrated in S14 Fig.

Nevertheless, docking experiments revealed that the predicted active sites of the Axitinib and SDC ligand, as well as the identified protein receptors in the breast and ovarian receptors, exhibited a higher binding affinity for SDC than axitinib alone. Axitinib and SDC demonstrated a higher affinity for binding to Caspase-8 protein receptors, with a value of (−6.6) and −7.7 kcal/mol, respectively (S15 Fig). The potential interaction mechanism between Axitinib and SDC comparison efficacy was determined by investigating the active site of a diverse array of breast and ovarian cancer proteins that were specifically targeted using molecular docking.

This study’s findings suggest that Axitinib and SDC interact with the targeted and selected protein receptor (Caspase-8 protein) on breast and ovarian cancer through a variety of hydrogen, conventional, covalent, and pi alkyl bonding interactions. A number of interactions between overrepresented protein domains in the network were revealed by the functional enrichment analysis, as the pathway of determination dictated the interaction categories. By this, it may be inferred that the system is presently operating with specific biological functions or pathways. Hydrophobic and H-bonding interactions are prevalent in the majority of complexes. S16 Fig–S18 Fig illustrate the presence of H-bonding between the active side residues of the amide oxygen or nitrogen of the majority of compounds, albeit in a greater number of interactions, in contrast to Axitinib and SDC in 3D and 2D forms.

Protein classes in breast and ovarian cancer Caspase-8 receptors were evaluated for their principal identical interactions with axitinib and SDC. In addition to other binding affinities to other proteins, the primary mechanism of action for Axitinib and its combining with SDC for sustained release may be attributed to this binding affinity. Based on the findings of the investigation, SDC exhibited a significant affinity for the active sites of a diverse array of protein receptors that were identified, with an affinity degree that was superior to that of axitinib alone. The active site bound affinities of the Caspase-8 binding receptor for axitinib and SDC were −6.6% and −7.7 kcal/mol, respectively. The affinities for axitinib had been previously verified by Tayyab et al [63]. The ligand of active site 1 established alkyl bonds with the receptor over an average distance of angstroms, as demonstrated by additional investigations of the interactions with the active sites. Alkyl interactions in organic molecules are generally feeble and occur between homologous, nonreactive carbon groups. The intersection of the pi-electron density of the aromatic ring and the electron density of the alkyl group is the defining characteristic of pi-alkyl interactions between aromatic and aliphatic groups. These interactions suggest that a portion of the compound’s binding affinity may be attributed to strong hydrophobic bonds, non-covalent van der Waals interactions, and H. bonds. Hydrogen bonding, a type of weak interaction, is initiated by fluctuations in the electron density of atoms or molecules. The aforementioned interactions indicate that alpha guanine has the capacity to establish a stable complex with H. van der Waals forces and bonding.

A mechanistic approach involves the controlled release of a synergist compound to enhance the anticancer activity of axitinib in a manner that is more effective and targeted to specific cancer receptors or proteins in both ovarian and breast cancer types. SDC-incorporation with axitinib enhances the efficacy and promotes the bioactivity and adhesive performance in order to achieve this objective.

The widely-recognized method of developing innovative nanomedicines that are characterized by a high level of pharmacological efficacy and decreased adverse effects is the use of biomaterials to deliver traditional anticancer compounds. Furthermore, the encapsulation of lipophilic compounds in systems facilitates the modulation of their biopharmaceutical properties, thereby avoiding the necessity of organic solvents. SDC’s potential as a novel anticancer formulation was demonstrated by its physical entrapment with axitinib, which was characterized by high stability, in vitro anticancer efficacy, and increased affinity in molecular docking investigations conducted in our study. It is evident that there are numerous obstacles to effective clinical translation that must be resolved, including both the delivery (e.g., biological challenges) and regulatory aspects (e.g., study, design, and approval challenges) [64].

Sodium glycocholate (SGC), sodium deoxycholate (SDC), and sodium taurocholate (STC) are the most frequently employed bile salts in the production of bilosomes. The primary reason for the selection of sodium glycocholate (SGC), sodium deoxycholate (SDC), and sodium taurocholate (STC) as the primary bile salts in bilosomes is their distinctive properties and advantages in the drug delivery system [65,66].

The apoptotic functions of caspase-8 are widely recognized, and it is an aspartate-specific cysteine protease. The central centers of multiple signal pathways are the locations of Caspase-8, which is responsible for regulating the cell cycle, invasive and metastatic cell behavior, immune cell homeostasis, and cytokine secretion. These are the two primary components of the tumor microenvironment. Caspase-8 expression is frequently dysregulated in ovarian cancer, leading to a discrepancy between its apoptotic and non-apoptotic functions in the tumor and the surrounding environment. According to the downregulation of caspase-8, ovarian cancer is linked to high aggressiveness, chronic inflammation, immunoediting, and immune resistance. Caspase-8 is therefore essential for the immune response, B and T lymphocyte activation, and macrophage differentiation and polarization in the TME, in addition to the primary tumor cells.

Consequently, In malignancies, caspase-8 exerts a dual function by modulating the reorganization of the tumor microenvironment and the expression profile of the tumor. Further investigation is required to determine whether caspase-8 regulates the inflammatory tumor milieu in favor of tumor promotion or suppression in order to evaluate its clinical relevance as a modulator of the tumor microenvironment. This information would provide us with supplementary valuable information about the modified factors by Caspase-8, which could be used as new pharmacological targets to reduce the tumor-supportive properties of the tumor microenvironment and to improve the tumor response to classical therapies [67].

The binding efficacy of axitinib and SDC, which have been experimentally bound to the receptors, is evaluated in the current study by calculating binding affinity and binding free energy (ΔG). The binding site and interaction were meticulously illustrated, and an exhaustive search was conducted to identify the factors that enhance these interactions and improve the binding efficacy. Consequently, the identification of potentially effective pharmaceuticals from the extensive pool of natural product and repurpose drug candidates is facilitated by a comprehension of the factors that reinforce these interactions, in addition to their binding sites and interactions. In determining the reactivity of the ligand-receptor binding, a computational chemistry, molecular docking, or in silico toxicity investigation can be highly beneficial. Additionally, the binding sites and mechanisms of action that were identified.

Consequently, the aim of the molecular docking investigations conducted in this study was to precisely ascertain the mechanism of action and distinct pathway for the anticancer activity of the tested ligand. The purpose of this study was to evaluate the in vitro efficacy of axitinib against both types of cancer and to confirm a correlation between this effect and the inhibition of critical proteins that are crucial for the survival of cancer cells.

In conclusion, our investigation suggests that Axitinib and SDC exhibit anti-breast and anti-ovarian cancer activity. The presence of SDC will enhance this activity in a controlled manner, influencing genes that are involved in the cell cycle, apoptosis, oxidation, and cell function of cancer cells. This will affect a variety of proteins. In order to assess the potential of Axitinib and SDC as a therapeutic strategy for cancer therapy and to develop a comprehensive comprehension of the molecular mechanisms that underlie their anticancer activity, further research is required. The inhibition affinity (pKd and ΔG) of Axitinib and SDC were assessed in association with the cancer functions receptor, which was recently crystallized through X-ray diffraction. The results suggested that Axitinib and SDC exhibited a significantly higher affinity for the receptor. The analysis revealed that the active site contained the following amino acid residues that were crucial for binding: (as determined. Hydrophobic interactions and hydrogen bonding are components of interactions. Furthermore, it has been established that Axitinib and SDC may act as a synergistic agent in conjunction with other chemotherapy agents to improve efficacy. It is possible to infer that Axitinib and SDC reduced the activity of the caspase-8 receptor on the surface of breast and ovarian cancer, which in turn resulted in an increase in anticancer activity, as indicated by molecular docking.

## 6. Conclusions

The central composite rotatable design showed success in optimizing AXT BSMs with the goal of minimizing VS and ZP while maximizing EE%. The optimum bilosomal formula displayed adequate VS, surface charge, and AXT loading. TEM image indicated spherical, non-aggregating vesicles that were stable physically for 30 days. When compared to AXT suspension, the optimal formula enhanced the drug release. The encapsulation of AXT within the bilosomal vesicles was confirmed by DSC and XRD measurements. Cell proliferation assays on MCF-7 breast and OV-2774 ovarian cancer cell lines revealed that optimal AXT-loaded BSMs were more effective than AXT suspension in lowering cell survival, even at lower concentrations. Flow cytometry revealed that AXT-loaded BSMs significantly increased the proportion of apoptotic cells in MCF-7 and OV-2774 cancer cell lines in comparison with free drug. Molecular docking suggests that axitinib and SDC decreased the activation of the caspase-8 receptor on the surface of ovarian and breast cancer, which consequently led to an increase in anticancer activity. As a result, AXT-loaded BSMs could be considered as a promising carrier for the treatment of breast and ovarian cancers.

## Supporting information

S1 Fig (A) 3D surface plot, (B) cube graph, (C) contour plot for the effect of.
(DOCX)

S2 Fig (A) 3D surface plot, (B) cube graph, (C) contour plot for the effect of independent variables on the VS of AXT loaded BSMs.
(DOCX)

S3 Fig (A) 3D surface plot, (B) cube graph, (C) contour plot for the effect of independent variables on ZP of AXT loaded BSMs.
(DOCX)

S4 Fig Numerical optimization and bar graph for AXT loaded BSMs using Central Composite design. (DOCX)

S5 Fig TEM image of the optimum AXT loaded BSMs. (DOCX)

S6 Fig In-vitro release profile of AXT from optimum bilosomal formula compared to AXT suspension. (DOCX)

S7 Fig Stability study for the optimized BSMs formula for 30 days at 4°C. (DOCX)

S8 Fig DSC thermograms of (A) pure AXT; (B) cholesterol, span60, SDC, and AXT physical mixture; (C) optimum AXT loaded BSMs.
(DOCX)

S9 Fig XRD pattern of (A) pure AXT; (B) cholesterol, span60, SDC, and AXT physical mixture; (C) optimum AXT loaded BSMs.
(DOCX)

S10 Fig Cytotoxicity of plain-BSMs, free AXT and AXT-BSMs in cancer cells. (A) **MCF-7 cells were treated with 0.4-fold serial dilution increase in all AXT formulation (0.4 µM–1.6 μM).** (B) OV-2774 cells were treated with 10-fold serial dilution increase in AXT loaded BSMs (10 µM–40 μM). The WST-1 assay was used to investigate the effects of AXT in different dosage form on the cell viability. Cell viability was expressed as a percentage of live cells relative to 0 µm of the treatment. Medications showed a concentration-dependent reduction in cell viability. The comparisons between groups were analysed using one way analysis of variance (ANOVA). Analysis was performed using GraphPad Prism 9. Results were expressed as mean ± standard deviation (SD). * *p* < 0.05 versus free drug.
(DOCX)

S11 Fig Effect of AXT BSMs formulation on cancer apoptosis induction. (A) **MCF-7 cells were treated with (1.1 μM) for both plain BSMs and free drug, (0.7 μM) for AXT loaded BSMs for 72 h. And** (B) **OV-2774 cells were treated with (36 μM) of plainBSMs, free drug, and AXTBSMs nano-formula for 72 hrs. Treated cells were stained with Annexin V and PI and analyzed by flow cytometry.** (C) The percentage of apoptotic cells was significantly higher in AXTBSMs compared to free drug suspension (74.79% vs. 55.15%) in MCF-7 and (52.2 vs 38.23) in OV-2774. The comparisons between groups were analysed using one way analysis of variance (ANOVA). Analysis was performed using Microsoft Office Excel 2016 and GraphPad Prism 9. Results were expressed as mean ± standard deviation (SD). * *p* < 0.05, ** *p* < 0.01 and *** *p* < 0.001 versus free drug.
(DOCX)

S12 Fig Caspase-8 Receptor.pdb.id & chemical structure (3KJQ) Structure, ID code and origin or source of protein with the shared amino acids. (DOCX)

S13 Fig Pocket, target of Caspase-8 protein and its shared amino acids in the active site of binding with axitinib. (DOCX)

S14 Fig Grid box centres and dimensions for Caspase-8 protein, for both ligands Axitinib and SDC. (DOCX)

S15 Fig Binding affinity (kcal/mol) scoring degree of binding and interactions between Axitinib& SDC on Caspase-8 protein receptors. (DOCX)

S16 Fig 3D &2D docking interactions sites between Axitinib and Caspase-8. (DOCX)

S17 Fig Shared amino acids at interactions sites between Axitinib and Caspase-8. (DOCX)

S18 Fig 3D &2D docking interactions sites between SDC and Caspase-8. (DOCX)

## Abbreviations

AXT: Axitinib
SDC: Sodium deoxy cholate
EE%: Entrapment efficiency
VS: Vesicles’ size
ZP: Zeta potential
XRD: X-ray diffraction
TEM: Transmission electron microscope
BSMs: Bilosomes
HER-2: Epidermal growth factor receptor-2
VEGF: Vascular endothelial growth factor
PDGF: Platelet derived growth factor
MCF-7: Michigan Cancer Foundation-7 (Breast cancer cell line)
OV-2774: Ovarian cancer cell line 2774
ATCC: American Type Culture Collection
DMEM/F12: Dulbecco’s Modified Eagle Medium/Nutrient Mixture F-12
FBS: Fetal Bovine Serum
CO2: Carbon Dioxide
WST1: Tetrazolium Salt
ELISA: Enzyme-Linked Immunosorbent Assay
IC50: Half-Maximal Inhibitory Concentration
PBS: Phosphate Buffered Solution
PI: Propidium Iodide
ANOVA: One Way Analysis of Variance
SD: Standard Deviation

## References

[1] Jin C, Wang K, Oppong-Gyebi A, Hu J. Application of nanotechnology in cancer diagnosis and therapy - A mini-review. Int J Med Sci. 2020;17(18):2964–73. doi: 10.7150/ijms.49801 33173417 PMC7646098

[2] Zaki RM, Alkharashi LA, Sarhan OM, Almurshedi AS, Aldosari BN, Said M. Box Behnken optimization of cubosomes for enhancing the anticancer activity of metformin: Design, characterization, and in-vitro cell proliferation assay on MDA-MB-231 breast and LOVO colon cancer cell lines. Int J Pharm X. 2023;6:100208. doi: 10.1016/j.ijpx.2023.100208 37680878 PMC10480553

[3] Mathews PD, Mertins O, Angelov B, Angelova A. Cubosomal lipid nanoassemblies with pH-sensitive shells created by biopolymer complexes: A synchrotron SAXS study. J Colloid Interface Sci. 2022;607:440–50. 34509118 10.1016/j.jcis.2021.08.187

[4] Dorling L, Carvalho S, Allen J, Gonzalez-Neira A, Luccarini C, Wahlström C. Breast cancer risk genes-association analysis in more than 113,000 women. N Engl J Med. 2021;384(5):428–39. 33471991 10.1056/NEJMoa1913948 PMC7611105

[5] Hussain Z, Khan JA, Murtaza S. Nanotechnology: An emerging therapeutic option for breast cancer. Criti Rev Eukaryot Gene Expr. 2018;28(2). 10.1615/CritRevEukaryotGeneExpr.2018022771 30055543

[6] Mendes LP, Sarisozen C, Torchilin VP. Physiological barriers in cancer: a challenge to be overcome. Functional Lipid Nanosystems in Cancer: Jenny Stanford Publishing; 2021. p. 3–43.

[7] Annaji M, Poudel I, Boddu SHS, Arnold RD, Tiwari AK, Babu RJ. Resveratrol-loaded nanomedicines for cancer applications. Cancer Rep (Hoboken). 2021;4(3):e1353. doi: 10.1002/cnr2.1353 33655717 PMC8222557

[8] Gatenby RA, Brown JS. Integrating evolutionary dynamics into cancer therapy. Nat Rev Clin Oncol. 2020;17(11):675–86. doi: 10.1038/s41571-020-0411-1 32699310

[9] Shen SF, Zhu LF, Liu J, Ali A, Zaman A, Ahmad Z. Novel core-shell fiber delivery system for synergistic treatment of cervical cancer. J Drug Deliv Sci Technol. 2020;59:101865.

[10] Bhattacharya S, Anjum MM, Patel KK. Gemcitabine cationic polymeric nanoparticles against ovarian cancer: formulation, characterization, and targeted drug delivery. Drug Deliv. 2022;29(1):1060–74. doi: 10.1080/10717544.2022.2058645 35363113 PMC8979509

[11] Cazzaniga ME, Giordano M, Bandera M, Cassani C, Bounous V, Lania A, et al. Managing menopausal symptoms in young women with breast cancer: When medicine is not all. The take care project. Clin Breast Cancer. 2021;21(5):e547–60. doi: 10.1016/j.clbc.2021.01.010 33685833

[12] Choi JY, Ramasamy T, Kim SY, Kim J, Ku SK, Youn YS, et al. PEGylated lipid bilayer-supported mesoporous silica nanoparticle composite for synergistic co-delivery of axitinib and celastrol in multi-targeted cancer therapy. Acta Biomater. 2016;39:94–105. doi: 10.1016/j.actbio.2016.05.012 27163403

[13] Ganesh K, Stadler ZK, Cercek A, Mendelsohn RB, Shia J, Segal NH, et al. Immunotherapy in colorectal cancer: rationale, challenges and potential. Nat Rev Gastroenterol Hepatol. 2019;16(6):361–75. 30886395 10.1038/s41575-019-0126-x PMC7295073

[14] Xie Y-H, Chen Y-X, Fang J-Y. Comprehensive review of targeted therapy for colorectal cancer. Signal Transduct Target Ther. 2020;5(1):22. doi: 10.1038/s41392-020-0116-z 32296018 PMC7082344

[15] Manzanares-Guevara LA, Licea-Claverie A, Oroz-Parra I, Bernaldez-Sarabia J, Diaz-Castillo F, Licea-Navarro AF. Smart nanoformulation based on stimuli-responsive nanogels and curcumin: Promising therapy against colon cancer. ACS Omega. 2020;5(16):9171–84. doi: 10.1021/acsomega.9b04390 32363269 PMC7191563

[16] Idoudi S, Bedhiafi T, Sahir F, Hijji Y, Uddin S, Merhi M, et al. Studies on anti-colon cancer potential of nanoformulations of curcumin and succinylated curcumin in mannosylated chitosan. Int J Biol Macromol. 2023;235:123827. doi: 10.1016/j.ijbiomac.2023.123827 36858085

[17] Hu J, Huang W, Huang S, ZhuGe Q, Jin K, Zhao Y. Magnetically active Fe 3 O 4 nanorods loaded with tissue plasminogen activator for enhanced thrombolysis. Nano Res. 2016;9:2652–61.

[18] Hu J, Huang S, Zhu L, Huang W, Zhao Y, Jin K, et al. Tissue plasminogen activator-porous magnetic microrods for targeted thrombolytic therapy after ischemic stroke. ACS Appl Mater Interfaces. 2018;10(39):32988–97. doi: 10.1021/acsami.8b09423 30192506

[19] Chaturvedi VK, Singh A, Singh VK, Singh MP. Cancer nanotechnology: A new revolution for cancer diagnosis and therapy. Curr Drug Metab. 2019;20(6):416–29. doi: 10.2174/1389200219666180918111528 30227814

[20] Choi JY, Ramasamy T, Tran TH, Ku SK, Shin BS, Choi H-G, et al. Systemic delivery of axitinib with nanohybrid liposomal nanoparticles inhibits hypoxic tumor growth. J Mater Chem B. 2015;3(3):408–16. doi: 10.1039/c4tb01442a 32262043

[21] Xu X, Li L, Zhou Z, Sun W, Huang Y. Dual-pH responsive micelle platform for co-delivery of axitinib and doxorubicin. Int J Pharm. 2016;507(1–2):50–60. doi: 10.1016/j.ijpharm.2016.04.060 27154256

[22] Ji M, Liu H, Wang H, Liang X, Wei M, Shi D, et al. pH-Activatable copper-axitinib coordinated multifunctional nanoparticles for synergistic chemo-chemodynamic therapy against aggressive cancers. Biomater Sci. 2023;11(18):6267–79. doi: 10.1039/d3bm00861d 37545202

[23] Almoshari Y. Development, therapeutic evaluation and theranostic applications of cubosomes on cancers: An updated review. Pharmaceutics. 2022;14(3):600. doi: 10.3390/pharmaceutics14030600 35335975 PMC8954425

[24] Saifi Z, Rizwanullah M, Mir SR, Amin S. Bilosomes nanocarriers for improved oral bioavailability of acyclovir: A complete characterization through in vitro, ex-vivo and in vivo assessment. J Drug Deliv Sci Technol. 2020;57:101634.

[25] Grasso M, Caruso G, Godos J, Bonaccorso A, Carbone C, Castellano S. Improving cognition with nutraceuticals targeting tgf-β1 signaling. Antioxidants. 2021;10(7):1075. 34356309 10.3390/antiox10071075 PMC8301008

[26] Ahmad J, Singhal M, Amin S, Rizwanullah M, Akhter S, Kamal MA, et al. Bile salt stabilized vesicles (Bilosomes): A novel nano-pharmaceutical design for oral delivery of proteins and peptides. Curr Pharm Des. 2017;23(11):1575–88. doi: 10.2174/1381612823666170124111142 28120725

[27] Shukla A, Mishra V, Kesharwani P. Bilosomes in the context of oral immunization: development, challenges and opportunities. Drug Discov Today. 2016;21(6):888–99. doi: 10.1016/j.drudis.2016.03.013 27038539

[28] Al-Mahallawi AM, Abdelbary AA, Aburahma MH. Investigating the potential of employing bilosomes as a novel vesicular carrier for transdermal delivery of tenoxicam. Int J Pharm. 2015;485(1–2):329–40. doi: 10.1016/j.ijpharm.2015.03.033 25796122

[29] Alhakamy NA, Badr-Eldin SM, Alharbi WS, Alfaleh MA, Al-Hejaili OD, Aldawsari HM, et al. Development of an icariin-loaded bilosome-melittin formulation with improved anticancer activity against cancerous pancreatic cells. Pharmaceuticals (Basel). 2021;14(12):1309. doi: 10.3390/ph14121309 34959710 PMC8703505

[30] Alhakamy NA, Caruso G, Al-Rabia MW, Badr-Eldin SM, Aldawsari HM, Asfour HZ, et al. Piceatannol-loaded bilosome-stabilized zein protein exhibits enhanced cytostatic and apoptotic activities in lung cancer cells. Pharmaceutics. 2021;13(5):638. doi: 10.3390/pharmaceutics13050638 33947103 PMC8146359

[31] Hegazy H, Amin MM, Fayad W, Zakaria MY. TPGS surface modified bilosomes as boosting cytotoxic oral delivery systems of curcumin against doxorubicin resistant MCF-7 breast cancer cells. Int J Pharm. 2022;619:121717. doi: 10.1016/j.ijpharm.2022.121717 35378174

[32] Zaki I, Abou-Elkhair RAI, Abu Almaaty AH, A Abu Ali O, Fayad E, Ahmed Gaafar AG, et al. Design and synthesis of newly synthesized acrylamide derivatives as potential chemotherapeutic agents against MCF-7 breast cancer cell line lodged on PEGylated bilosomal nano-vesicles for improving cytotoxic activity. Pharmaceuticals (Basel). 2021;14(10):1021. doi: 10.3390/ph14101021 34681245 PMC8540948

[33] Youness RA, Al-Mahallawi AM, Mahmoud FH, Atta H, Braoudaki M, Fahmy SA. Oral delivery of psoralidin by mucoadhesive surface-modified bilosomes showed boosted apoptotic and necrotic effects against breast and lung cancer cells. Polymers (Basel). 2023;15(6):1464. doi: 10.3390/polym15061464 36987244 PMC10052996

[34] Alamoudi AJ, Badr-Eldin SM, Ahmed OAA, Fahmy UA, Elbehairi SEI, Alfaifi MY, et al. Optimized bilosome-based nanoparticles enhance cytotoxic and pro-apoptotic activity of costunolide in LS174T colon cancer cells. Biomed Pharmacother. 2023;168:115757. doi: 10.1016/j.biopha.2023.115757 37897972

[35] Abou Assi R, Abdulbaqi IM, Tan SM, Wahab HA, Darwis Y, Chan S-Y. Breaking barriers: bilosomes gel potentials to pave the way for transdermal breast cancer treatment with Tamoxifen. Drug Dev Ind Pharm. 2023:1–12. doi: 10.1080/03639045.2023.2256404 37722711

[36] Kaurav H, Tripathi M, Kaur SD, Bansal A, Kapoor DN, Sheth S. Emerging trends in bilosomes as therapeutic drug delivery systems. Pharmaceutics. 2024;16(6):697. 38931820 10.3390/pharmaceutics16060697 PMC11206586

[37] Nemr AA, El-Mahrouk GM, Badie HA. Hyaluronic acid-enriched bilosomes: an approach to enhance ocular delivery of agomelatine via D-optimal design: formulation, in vitro characterization, and in vivo pharmacodynamic evaluation in rabbits. Drug Deliv. 2022;29(1):2343–56. doi: 10.1080/10717544.2022.2100513 35869684 PMC9477486

[38] Zaki RM, Alfadhel MM, Alossaimi MA, Elsawaf LA, Devanathadesikan Seshadri V, Almurshedi AS, et al. Central composite optimization of glycerosomes for the enhanced oral bioavailability and brain delivery of quetiapine fumarate. Pharmaceuticals (Basel). 2022;15(8):940. doi: 10.3390/ph15080940 36015089 PMC9412614

[39] Zaki RM, Seshadri VD, Mutayran AS, Elsawaf LA, Hamad AM, Almurshedi AS, et al. Wound healing efficacy of rosuvastatin transethosomal Gel, I optimal optimization, histological and in vivo evaluation. Pharmaceutics. 2022;14(11):2521. doi: 10.3390/pharmaceutics14112521 36432712 PMC9692372

[40] Mazyed EA, Abdelaziz AE. Fabrication of transgelosomes for enhancing the ocular delivery of acetazolamide: Statistical optimization, in vitro characterization, and in vivo study. Pharmaceutics. 2020;12(5):465. doi: 10.3390/pharmaceutics12050465 32443679 PMC7284610

[41] Salem HF, Kharshoum RM, Abou-Taleb HA, Farouk HO, Zaki RM. Fabrication and appraisal of simvastatin via tailored niosomal nanovesicles for transdermal delivery enhancement: In vitro and in vivo assessment. Pharmaceutics. 2021;13(2):138. doi: 10.3390/pharmaceutics13020138 33494472 PMC7910921

[42] Hosseinzadeh H, Atyabi F, Dinarvand R, Ostad SN. Chitosan-Pluronic nanoparticles as oral delivery of anticancer gemcitabine: preparation and in vitro study. Int J Nanomedicine. 2012;7:1851–63. doi: 10.2147/IJN.S26365 22605934 PMC3352690

[43] Salem HF, Kharshoum RM, Mahmoud M, Azim SA, Ebeid E-ZM. Development and characterization of a novel nano-liposomal formulation of Alendronate Sodium loaded with biodegradable polymer. Ars Pharmaceutica. 2018;59(1):9–20.

[44] de Sá FAP, Taveira SF, Gelfuso GM, Lima EM, Gratieri T. Liposomal voriconazole (VOR) formulation for improved ocular delivery. Colloids Surf B: Biointerfaces. 2015;133:331–8. 26123854 10.1016/j.colsurfb.2015.06.036

[45] Aboud HM, Mahmoud MO, Abdeltawab Mohammed M, Shafiq Awad M, Sabry D. Preparation and appraisal of self-assembled valsartan-loaded amalgamated Pluronic F127/Tween 80 polymeric micelles: Boosted cardioprotection via regulation of Mhrt/Nrf2 and Trx1 pathways in cisplatin-induced cardiotoxicity. J Drug Target. 2020;28(3):282–99. doi: 10.1080/1061186X.2019.1650053 31353972

[46] Said M, Aboelwafa AA, Elshafeey AH, Elsayed I. Central composite optimization of ocular mucoadhesive cubosomes for enhanced bioavailability and controlled delivery of voriconazole. J Drug Deliv Sci Technol. 2021;61:102075.

[47] Said M, Elsayed I, Aboelwafa AA, Elshafeey AH. Transdermal agomelatine microemulsion gel: pyramidal screening, statistical optimization and in vivo bioavailability. Drug Deliv. 2017;24(1):1159–69. doi: 10.1080/10717544.2017.1365392 28831842 PMC8241019

[48] Said M, Elsayed I, Aboelwafa AA, Elshafeey AH. A novel concept of overcoming the skin barrier using augmented liquid nanocrystals: Box-Behnken optimization, ex vivo and in vivo evaluation. Colloids Surf B: Biointerfaces. 2018;170:258–65. 29935419 10.1016/j.colsurfb.2018.06.025

[49] Zafar A, Alruwaili NK, Imam SS, Hadal Alotaibi N, Alharbi KS, Afzal M, et al. Bioactive Apigenin loaded oral nano bilosomes: Formulation optimization to preclinical assessment. Saudi Pharm J. 2021;29(3):269–79. doi: 10.1016/j.jsps.2021.02.003 33981176 PMC8085606

[50] Ameeduzzafar, Alruwaili NK, Imam SS, Alotaibi NH, Alhakamy NA, Alharbi KS, et al. Formulation of chitosan polymeric vesicles of ciprofloxacin for ocular delivery: Box-behnken optimization, in vitro characterization, HET-CAM irritation, and antimicrobial assessment. AAPS PharmSciTech. 2020;21(5):167. doi: 10.1208/s12249-020-01699-9 32504176

[51] Badria F, Mazyed E. Formulation of nanospanlastics as a promising approach for improving the topical delivery of a natural leukotriene inhibitor (3-acetyl-11-keto-β-boswellic acid): Statistical optimization, in vitro characterization, and ex vivo permeation study. Drug Des Devel Ther. 2020:3697–721. 10.2147/DDDT.S265167 PMC7501970 32982176

[52] Salem HF, Nafady MM, Ali AA, Khalil NM, Elsisi AA. Evaluation of metformin hydrochloride tailoring bilosomes as an effective transdermal nanocarrier. Int J Nanomedicine. 2022;17:1185–201. doi: 10.2147/IJN.S345505 35330695 PMC8938169

[53] Opatha SAT, Titapiwatanakun V, Chutoprapat R. Transfersomes: A promising nanoencapsulation technique for transdermal drug delivery. Pharmaceutics. 2020;12(9):855. doi: 10.3390/pharmaceutics12090855 32916782 PMC7559928

[54] Dave V, Yadav RB, Kushwaha K, Yadav S, Sharma S, Agrawal U. Lipid-polymer hybrid nanoparticles: Development & statistical optimization of norfloxacin for topical drug delivery system. Bioact Mater. 2017;2(4):269–80. doi: 10.1016/j.bioactmat.2017.07.002 29744436 PMC5935510

[55] Farmoudeh A, Akbari J, Saeedi M, Ghasemi M, Asemi N, Nokhodchi A. Methylene blue-loaded niosome: preparation, physicochemical characterization, and in vivo wound healing assessment. Drug Deliv Transl Res. 2020;10(5):1428–41. doi: 10.1007/s13346-020-00715-6 32100265 PMC7447683

[56] Owodeha-Ashaka K, Ilomuanya MO, Iyire A. Evaluation of sonication on stability-indicating properties of optimized pilocarpine hydrochloride-loaded niosomes in ocular drug delivery. Prog Biomater. 2021;10:207–20. 34549376 10.1007/s40204-021-00164-5 PMC8511210

[57] Ismail A, Teiama M, Magdy B, Sakran W. Development of a novel bilosomal system for improved oral bioavailability of sertraline hydrochloride: Formulation design, in vitro characterization, and ex vivo and in vivo studies. AAPS PharmSciTech. 2022;23(6):188. doi: 10.1208/s12249-022-02339-0 35799076

[58] Dehghani F, Farhadian N, Golmohammadzadeh S, Biriaee A, Ebrahimi M, Karimi M. Preparation, characterization and in-vivo evaluation of microemulsions containing tamoxifen citrate anti-cancer drug. Eur J Pharm Sci. 2017;96:479–89. doi: 10.1016/j.ejps.2016.09.033 27693298

[59] Salem HF, Kharshoum RM, Abdel Hakim LF, Abdelrahim ME. Edge activators and a polycationic polymer enhance the formulation of porous voriconazole nanoagglomerate for the use as a dry powder inhaler. J Liposome Res. 2016;26(4):324–35. doi: 10.3109/08982104.2016.1140182 26872552

[60] Salem HF, Kharshoum RM, Sayed OM, Abdel Hakim LF. Formulation design and optimization of novel soft glycerosomes for enhanced topical delivery of celecoxib and cupferron by Box–Behnken statistical design. Drug Dev Ind Pharm. 2018;44(11):1871–84. 30044654 10.1080/03639045.2018.1504963

[61] Halasz K, Kelly SJ, Iqbal MT, Pathak Y, Sutariya V. Utilization of apatinib-loaded nanoparticles for the treatment of ocular neovascularization. Curr Drug Deliv. 2019;16(2):153–63. doi: 10.2174/1567201815666181017095708 30332959

[62] Tayyab S, Izzudin MM, Kabir MZ, Feroz SR, Tee W-V, Mohamad SB, et al. Binding of an anticancer drug, axitinib to human serum albumin: Fluorescence quenching and molecular docking study. J Photochem Photobiol B. 2016;162:386–94. doi: 10.1016/j.jphotobiol.2016.06.049 27424099

[63] Narvekar P, Bhatt P, Fnu G, Sutariya V. Axitinib-loaded poly (lactic-co-glycolic acid) nanoparticles for age-related macular degeneration: formulation development and in vitro characterization. Assay Drug Dev Technol. 2019;17(4):167–77. 31184962 10.1089/adt.2019.920

[64] Gagliardi A, Bonacci S, Paolino D, Celia C, Procopio A, Fresta M, et al. Paclitaxel-loaded sodium deoxycholate-stabilized zein nanoparticles: characterization and in vitro cytotoxicity. Heliyon. 2019;5(9):e02422. doi: 10.1016/j.heliyon.2019.e02422 31517130 PMC6734341

[65] Ge X, Wei M, He S, Yuan W-E. Advances of non-ionic surfactant vesicles (Niosomes) and their application in drug delivery. Pharmaceutics. 2019;11(2):55. doi: 10.3390/pharmaceutics11020055 30700021 PMC6410054

[66] Wilkhu JS, McNeil SE, Anderson DE, Perrie Y. Characterization and optimization of bilosomes for oral vaccine delivery. J Drug Target. 2013;21(3):291–9. doi: 10.3109/1061186X.2012.747528 30952177

[67] Kostova I, Mandal R, Becker S, Strebhardt K. The role of caspase-8 in the tumor microenvironment of ovarian cancer. Cancer Metastasis Rev. 2021;40(1):303–18. doi: 10.1007/s10555-020-09935-1 33026575 PMC7897206

