# Supplementary material for: Fabrication and appraisal of targeted axitinib loaded bilosomes for the enhanced breast and ovarian anticancer activity
Source: PLoS One. 2025 Jul 17;20(7):e0325511. doi: 10.1371/journal.pone.0325511 (PMC12270130; doi:10.1371/journal.pone.0325511)
Supplement: S2 Fig — (A) 3D surface plot, (B) cube graph, (C) contour plot for the effect of independent variables on the VS of AXT loaded BSMs. (DOCX) [file pone.0325511.s002.docx]

**S2 Fig. (A)3D surface plot, (B) cube graph, (C) contour plot for the effect of independent variables on the VS of AXT loaded BSMs.**
