# Supplementary material for: Fabrication and appraisal of targeted axitinib loaded bilosomes for the enhanced breast and ovarian anticancer activity
Source: PLoS One. 2025 Jul 17;20(7):e0325511. doi: 10.1371/journal.pone.0325511 (PMC12270130; doi:10.1371/journal.pone.0325511)
Supplement: S9 Fig — XRD pattern of (A) pure AXT; (B) cholesterol, span60, SDC, and AXT physical mixture; (C) optimum AXT loaded BSMs. (DOCX) [file pone.0325511.s009.docx]

**S9 Fig. XRD pattern of (A) pure AXT; (B) cholesterol, span60, SDC, and AXT physical mixture; (C) optimum AXT loaded BSMs.**
