# Supplementary material for: Fabrication and appraisal of targeted axitinib loaded bilosomes for the enhanced breast and ovarian anticancer activity
Source: PLoS One. 2025 Jul 17;20(7):e0325511. doi: 10.1371/journal.pone.0325511 (PMC12270130; doi:10.1371/journal.pone.0325511)
Supplement: S10 Fig — (A) MCF-7 cells were treated with 0.4-fold serial dilution increase in all AXT formulation (0.4 µM–1.6 μM). (B) OV-2774 cells were treated with 10-fold serial dilution increase in AXT loaded BSMs (10 µM–40 μM). The WST-1 assay was used to investigate the effects of AXT in different dosage form on the cell viability. Cell viability was expressed as a percentage of live cells relative to 0 µm of the treatment. Medications showed a concentration-dependent reduction in cell viability. The comparisons between groups were analysed using one way analysis of variance (ANOVA). Analysis was performed using GraphPad Prism 9. Results were expressed as mean ± standard deviation (SD). * p < 0.05 versus free drug. (DOCX) [file pone.0325511.s010.docx]

**S10 Fig Cytotoxicity of plain-BSMs, free AXT and AXT-BSMs in cancer cells. (A) MCF-7 cells were treated with 0.4-fold serial dilution increase in all AXT formulation (0.4 µM–1.6 μM). (B) OV-2774 cells were treated with 10-fold serial dilution increase in AXT loaded BSMs (10 µM–40 μM). The WST-1 assay was used to investigate the effects of AXT in different dosage form on the cell viability. Cell viability was expressed as a percentage of live cells relative to 0 µm of the treatment. Medications showed a concentration-dependent reduction in cell viability. The comparisons between groups were analysed using one way analysis of variance (ANOVA). Analysis was performed using GraphPad Prism 9. Results were expressed as mean ± standard deviation (SD). * *p* < 0.05 versus free drug.**
