# Supplementary material for: Fabrication and appraisal of targeted axitinib loaded bilosomes for the enhanced breast and ovarian anticancer activity
Source: PLoS One. 2025 Jul 17;20(7):e0325511. doi: 10.1371/journal.pone.0325511 (PMC12270130; doi:10.1371/journal.pone.0325511)
Supplement: S11 Fig — (A) MCF-7 cells were treated with (1.1 μM) for both plain BSMs and free drug, (0.7 μM) for AXT loaded BSMs for 72 h. And (B) OV-2774 cells were treated with (36 μM) of plainBSMs, free drug, and AXTBSMs nano-formula for 72 hrs. Treated cells were stained with Annexin V and PI and analyzed by flow cytometry. (C) The percentage of apoptotic cells was significantly higher in AXTBSMs compared to free drug suspension (74.79% vs. 55.15%) in MCF-7 and (52.2 vs 38.23) in OV-2774. The comparisons between groups were analysed using one way analysis of variance (ANOVA). Analysis was performed using Microsoft Office Excel 2016 and GraphPad Prism 9. Results were expressed as mean ± standard deviation (SD). * p < 0.05, ** p < 0.01 and *** p < 0.001 versus free drug. (DOCX) [file pone.0325511.s011.docx]

**S11 Fig. Effect of AXT BSMs formulation on cancer apoptosis induction. (A) MCF-7 cells were treated with (1.1 μM) for both plain BSMs and free drug, (0.7 μM) for AXT loaded BSMs for 72 h. And (B) OV-2774 cells were treated with (36 μM) of plainBSMs, free drug, and AXTBSMs nano-formula for 72 hrs. Treated cells were stained with Annexin V and PI and analyzed by flow cytometry. (C) The percentage of apoptotic cells was significantly higher in AXTBSMs compared to free drug suspension (74.79% vs. 55.15%) in MCF-7 and (52.2 vs 38.23) in OV-2774. The comparisons between groups were analysed using one way analysis of variance (ANOVA). Analysis was performed using Microsoft Office Excel 2016 and GraphPad Prism 9. Results were expressed as mean ± standard deviation (SD). * *p* < 0.05, ** *p* < 0.01 and *** *p* < 0.001 versus free drug.**
