# Supplementary material for: Fabrication and appraisal of targeted axitinib loaded bilosomes for the enhanced breast and ovarian anticancer activity
Source: PLoS One. 2025 Jul 17;20(7):e0325511. doi: 10.1371/journal.pone.0325511 (PMC12270130; doi:10.1371/journal.pone.0325511)
Supplement: S12 Fig — (DOCX) [file pone.0325511.s012.docx]

**S12 Fig. Caspase-8 Receptor.pdb.id & chemical structure (3KJQ) Structure, ID code and origin or source of protein with the shared amino acids.**
