# Supplementary material for: Fabrication and appraisal of targeted axitinib loaded bilosomes for the enhanced breast and ovarian anticancer activity
Source: PLoS One. 2025 Jul 17;20(7):e0325511. doi: 10.1371/journal.pone.0325511 (PMC12270130; doi:10.1371/journal.pone.0325511)
Supplement: S13 Fig — (DOCX) [file pone.0325511.s013.docx]

**S13 Fig. Pocket, target of Caspase-8 protein and its shared amino acids in the active site of binding with axitinib.**
