# Supplementary material for: Fabrication and appraisal of targeted axitinib loaded bilosomes for the enhanced breast and ovarian anticancer activity
Source: PLoS One. 2025 Jul 17;20(7):e0325511. doi: 10.1371/journal.pone.0325511 (PMC12270130; doi:10.1371/journal.pone.0325511)
Supplement: S14 Fig — (DOCX) [file pone.0325511.s014.docx]

**S14 Fig. Grid box centres and dimensions for Caspase-8 protein, for both ligands Axitinib and SDC.**
