# Supplementary material for: Fabrication and appraisal of targeted axitinib loaded bilosomes for the enhanced breast and ovarian anticancer activity
Source: PLoS One. 2025 Jul 17;20(7):e0325511. doi: 10.1371/journal.pone.0325511 (PMC12270130; doi:10.1371/journal.pone.0325511)
Supplement: S17 Fig — (DOCX) [file pone.0325511.s017.docx]

**S17 Fig. Shared amino acids at interactions sites between Axitinib and Caspase-8.**
